# Developing culturally responsive sexual health interventions: recommendations from Middle Eastern and North African diaspora youth in Ontario, Canada

**DOI:** 10.3389/fpubh.2026.1912504

**Published:** 2026-09-09

**Authors:** Roula Kteily-Hawa, Vijaya Chikermane, Raed Hawa, Olesya Falenchuk, Bessma Momani, Praney Anand, Nadia N. Abuelezam, Rania Younes, Ahmad Ezzeddine, Nahla Abdel-Tawab, Mona Loutfy

**Affiliations:** 1 Faculty of Health Sciences, Western University, London, ON, Canada; 2 7.10 Stories, Toronto, ON, Canada; 3 Department of Psychiatry, University Health Network, Toronto, ON, Canada; 4 Ontario Institute for Studies in Education, University of Toronto, Toronto, ON, Canada; 5 Political Science, University of Waterloo, Waterloo, ON, Canada; 6 Alliance for South Asian Aids Prevention, Toronto, ON, Canada; 7 Michigan State University College of Human Medicine, Lansing, MI, United States; 8 OCAD University, Toronto, ON, Canada; 9 HIV & AIDS Legal Clinic Ontario (HALCO), Toronto, ON, Canada; 10 Population Council Egypt, Cairo, Egypt; 11 Women's College Hospital, Toronto, ON, Canada

**Keywords:** gay, bisexual and other men who have sex with men (GBM), community-based research, HIV and STBBI interventions, Middle Eastern and North African (MENA), lesbian, bisexual and queer women, trans and gender-diverse people (TGD), youth-young adults

## Abstract

**Introduction:**

Sex and sexual health practices of Middle Eastern and North African (MENA) youth in Canada are under-researched, even as the community grows rapidly due to high immigration rates. Through a community-based research approach, we examined access to sexual health services among MENA youth living in Ontario, Canada, and identified interventions to strengthen uptake of sexual health services.

**Methods:**

Fifty-six MENA youth were recruited through purposive and snowball sampling. Participants completed a brief sociodemographic survey and joined subgroups across five sexual and gender identity categories. Each group participated in two sequential dialogical focus groups, for a total of 14 sessions. This design supported relationship-building, iterative reflection and progressively deeper discussion. A final mixed focus group reconvened 12 participants from the earlier groups to refine intervention recommendations. Data were analyzed using collaborative codebook thematic analysis, with collaborative coding and negotiated interpretation among academic and peer researchers supporting analytic credibility.

**Results:**

Youth provided recommendations for behavioural, structural and biomedical interventions that reflected their community needs. Intervention suggestions common to all groups included culturally relevant and language-specific educational programs, online interactive resources leveraging social media, mentorship programs to facilitate community connection, and health navigation tools to strengthen sexual health access and service use. Interventions unique to specific subgroups were also offered. For example, trans women newcomers recommended anti-transphobia training with language interpreters, while gay and bisexual men recommended cost-free PrEP access.

**Discussion:**

Youth-driven interventions hold significant promise for strengthening the HIV and sexual health response among MENA youth in Canada. The knowledge generated by and with youth is especially relevant for community-based health organizations, healthcare practitioners and policymakers.

## Introduction

Young people and adolescents are disproportionately impacted by the global HIV epidemic. As of 2019, 1.6 million people between 10 and 19 years of age were living with HIV and 190,000 were newly infected (1). With a steadily growing youth population, the Middle East and North Africa (MENA) region is home to 80 million youth who make up 10% of the world’s population (2).

Today, the term ‘Middle East and North Africa’ or MENA is used by international organizations to refer to 23 countries in the region that share common socio-historic, linguistic and religious characteristics (e.g., Egypt, Iran, Iraq, Jordan, Lebanon, Syria, Yemen). Canada uses the term West Asian and Arab to refer to two distinct groups. For the purposes of the study and this paper, the term MENA is used to refer to first- or second-generation diaspora youth in Canada whose heritage lies among one or more of the countries in the MENA region.

According to the 2025 UNAIDS Global AIDS Update, between 2010 and 2024, the number of new HIV infections in the MENA region increased by about 94%, while in most other regions the numbers declined over the same period (3). A scoping review of the literature focused on epidemiological HIV risk factors and underlying risk context for youth residing in or originating from MENA found similar results (4). The increase in new HIV infections has mostly affected sex workers, people who inject drugs (PWID), and gay and other men who have sex with men (MSM), all of whom face harsh criminalization, stigma and discrimination (3, 4). Although AIDS-related deaths in the MENA region declined by approximately 6% over the same period, this modest improvement occurred alongside the sharply rising new infections and lagged behind broader global progress (3).

These global trends have implications in the Canadian landscape. According to Statistics Canada’s analysis of the 2021 Census, 62% of recent immigrants (2016–2021) were born in Asia, including the Middle East (5). Moreover, the number of immigrants from the MENA region are expected to more than triple in the next 25 years (2006-2031). With an influx of newcomer and refugee youth from the MENA region in Canada, many face unemployment, language barriers, limited social support systems, and cultural barriers (6), increasing their vulnerability to HIV (7). Youth, especially LGBTQ+ youth, immigrants, and refugees bear a disproportionate burden of STBBIs and HIV due to structural barriers impacting young people who experience social and economic marginalization (8).

These barriers must also be understood within the broader Canadian HIV context. HIV remains an unevenly distributed public health concern in Canada. In 2024, 2,288 new HIV diagnoses were reported nationally, representing a 3.3% increase since 2023 (9). Rates were considerably higher in Manitoba and Saskatchewan. Research in the Prairie provinces has linked these patterns to intersecting structural conditions, including stigma, housing insecurity, substance use, violence, and barriers to prevention and care, with disproportionate effects on marginalized communities (10). These patterns further underscore the importance of attending to intersecting structural inequities in HIV prevention and care.

Taken together, these intersecting considerations point to an important gap in Canadian research as there is limited research on the unique challenges that diverse youth from MENA communities face when accessing health and social services in the Canadian context (11), particularly around sexual health. Available literature typically leans towards MENA diaspora youth’s sense of home, belonging, acculturation, and identity in Canada, especially in Québec (11, 12). A scoping review on the sexual health of Muslim youth in Canada recommends the need for increased literature to support the development of effective health programming (13); however, this does not distinguish MENA youth from other Muslim youth in Canada or account for non-Muslim youth who identify from the MENA region. A wider selection of research is available in the United States that explores the health and sexual health realities of ‘Arab American’ youth, which can inform the study of Canadian MENA youth, although it is also sparse (14, 15). This study was particularly concerned with the dearth of knowledge of the sexual health-seeking behavior and experiences of MENA youth in a Canadian context.

Understanding the social determinants of health, risk behaviors, risk contexts, and lived experiences of youth from MENA communities in Canada is crucial to supporting the ongoing well-being of this growing community. In particular, participant inputs are important to the successful design and implementation of tailored and effective health interventions (16). In the HIV field peer-based education is considered a valuable strategy for effective health promotion (17). However, youth input is not typically sought in the development of health interventions (even less so for racialized, immigrant youth and those who identify with diverse sexual or gender identities) (18, 19).

The Youth Sexual Health Needs in Middle Eastern and North African Communities (YSMENA) study is a peer-led, community-based mixed-methods research program that addresses this gap in knowledge and explores experiences and perceptions of sexual health, HIV and other STBBIs of MENA youth in Ontario, Canada. YSMENA is the first community-based research study in Canada to center the voices of diaspora MENA youth. Within the broader project, this paper specifically examines how MENA youth across the sexual and gender spectrum understand and envision sexual health interventions, generating an in-depth, community-informed account of their priorities and recommendations. In accordance with community-based research, diverse MENA-identified youth were involved in varying aspects of the project and the research activities aimed to build youth capacity in sexual health knowledge generation.

This exploratory, community-based orientation was grounded in a critical constructivist paradigm, which understands knowledge as produced within sociohistorical contexts and relations of power (20). Research with sexual and gender minority people in MENA diaspora contexts demonstrates that sexual health experiences are shaped by intersecting cultural, gendered, social, and institutional conditions, including stigma, discrimination and access to contextually relevant information and services (6, 14). Accordingly, the sexual health perspectives of MENA youth were approached as situated forms of knowledge shaped by lived experience, cultural context and broader systems of power.

## Materials and methods

### Community-based research approach

Our main aim was to follow a research approach that emphasized the significance of collaboration, participation and social justice, with a focus on both individual MENA youth and the community as a whole. To that end, we drew on principles of community-based research (CBR), including meaningful community participation, recognition of community knowledge and strengths, reciprocal learning, capacity building, and the connection of knowledge generation with action (21, 22). These commitments were reflected in the formation of a 6-member community advisory committee (CAC), comprising 4 youth representatives of the MENA youth diaspora of diverse sexual identities and 2 adult MENA members of the community. Over a period of 1 year, members of the CAC met once in person and later conducted work via Zoom and email. CAC members provided input into the study survey, focus group discussion guide, and helped out in different tasks in the study, including interviewing for peer research associates and recruiting study participants.

The study’s peer-driven model provided another important avenue for youth participation. Peer-based approaches in HIV prevention and education are highly valued, with a key strength being that the intended audience can see relatable experiences in messaging and materials as these are developed by their peers (16). Accordingly, 6 Peer Research Associates (PRAs) from MENA diaspora communities and of diverse sexual identities were recruited to lead key aspects of the study. PRAs were required to complete extensive training including key principles of community and participatory research; research ethics and confidentiality; informed consent; managing difficult conversations; focus group facilitation methods; intersectionality; how to unpack privilege; and more. This investment in peer training and development was expected to enrich community engagement efforts and as research indicates, contribute to community capacity building (23–25).

Each focus group was facilitated by a team of 3 PRAs who served as either lead facilitator, co-facilitator, or field note-taker. Following each session, PRAs completed a descriptive and reflective journal to note their observations of the session. PRAs were compensated for their participation in training, data collection, and analysis activities. To support their well-being, they were informed that guidance was available through the psychiatrist and infectious disease physician on the research team and were provided with a list of relevant community-based psychosocial and health resources.

### Ethical considerations

A multi-jurisdictional ethics approval was obtained from University of Toronto’s Research Ethics Board and Brescia University College’s Research Ethics Board at the University of Western Ontario.

### Recruitment

Participants were recruited using purposive and snowball sampling. Purposive sampling involved circulating approved recruitment materials by email through trusted partner organizations and community networks likely to reach MENA youth who met the study’s eligibility criteria. Snowball recruitment allowed eligible participants and community contacts to share study information through word of mouth within their networks, supporting access to youth who may have been less readily reached through conventional recruitment channels because of stigma and privacy concerns (26). Interested youth contacted the study by completing an online screening survey. The survey confirmed whether individuals self-identified as Middle Eastern or North African, were between 16 and 38 years of age, lived in Ontario, were comfortable participating in English, had engaged in sexual activity during the previous year, and identified with one of the sexual and gender groups included in the study. The upper age limit was intentionally extended to 38 to reflect the lived realities of the communities involved and to adopt a more inclusive understanding of youth and young adulthood, recognizing that experiences and life transitions do not occur according to fixed age boundaries. Individuals who did not meet one or more of these criteria were excluded.

### Data collection

All participants were provided with a detailed letter of information and consent that they read and signed. A member of our research team was available via email or by phone, if needed, to answer any questions about the study. Following informed consent, data collection occurred in 3 phases. In Phase 1, participants completed a brief pre-focus group sociodemographic survey used to characterize the study sample. In Phase 2, participants completed an individual journal activity administered as an open-ended qualitative component of the survey. In Phase 3, participants took part in sequential dialogical focus groups. The sociodemographic survey was used descriptively, while the journal activity and focus groups generated the qualitative data for the study.

The journal activity served as an individual entry point into the qualitative discussions. It provided participants with an opportunity to reflect privately on potentially sensitive experiences before discussing related topics with others. Questions explored participants’ relationships and intimate lives, experiences accessing healthcare and sexual health services, and the ways their cultural and ethnic identities shaped their sexual experiences and practices. Prompts were adapted where appropriate to reflect participants’ sexual and gender identities.

Building on this individual reflection, participants then engaged in sequential dialogical focus groups. This approach requires engaging the same participants over separate sessions for discussion and dialogue. This method offers deeper engagement and reflection than a single-session focus group, and it is especially applicable to sensitive health subjects and/or marginalized populations (27).

A total of 56 participants were organized into 7 groups across 5 sexual and gender identity categories. These included 3 groups of gay and bisexual cisgender men and/or men who have sex with men and 1 group each of heterosexual cisgender men; heterosexual cisgender women; lesbian, bisexual, and queer cisgender women; and trans women. Each group participated in 2 focus group sessions resulting in 14 sessions. Together with the additional mixed focus group described below, 15 focus group sessions were conducted. All 15 focus group sessions were conducted virtually through Zoom and generally lasted 2 to 2.5 h. Sessions were audio-recorded with participants’ consent. Zoom-generated transcripts were reviewed against the audio recordings, corrected for accuracy, and finalized as verbatim transcripts before analysis. Table 1 summarizes the composition of the participant groups and the number of sessions conducted.

**Table 1:** Breakdown of the sessions held by population sub-group.

Population	# Of participants	# Of focus groups
Gay and/or MSM	22 participants across 3 groups	Each of the 3 groups underwent 2 focus groups for a total of 6 focus groups
Heterosexual women	11 participants in 1 group	2 focus groups held
Heterosexual men	12 participants in 1 group	2 focus groups held
Trans women	8 participants in 1 group	2 focus groups held, however 1 participant did not attend the second session
Lesbian, Bisexual and Queer (LBQ) women	3 participants in 1 group	2 focus groups held
Mixed group	12 returning participants (from above pool)	1 focus group held
Total # of sub-groups: 6	Total participants: 56	Total # of focus groups held: 15

The first focus group session explored the intersecting cultural, ethnic, sexual, gender, social, and structural factors shaping participants’ sexual health experiences and practices. Questions were adapted slightly across groups to reflect the identities represented and the contexts relevant to participants’ experiences.

Between the first and second focus group sessions, participants completed a take-home activity introducing 3 broad forms of sexual health intervention: behavioral, structural and biomedical. Behavioral interventions were defined as approaches focused on strengthening individuals’ knowledge and skills including interactive online education and peer or community mentorship programs. Structural interventions were defined as approaches intended to change the broader social, institutional or service conditions that shape sexual health and access to care including media-based initiatives and newcomer orientation programs. Biomedical interventions were defined as clinical or pharmaceutical approaches to preventing or identifying HIV and other STBBIs including testing and medications. Participants were asked to develop an “elevator pitch” for an intervention they believed would be relevant to them, their peers, or their communities. This activity provided another opportunity for individual reflection and prepared participants for the intervention-focused discussion in the second session.

The second focus group session examined barriers to accessing sexual health information and services and participants’ recommendations for behavioral, structural and biomedical interventions. Participants presented and discussed their proposed interventions and considered their relevance, importance and practical application. Table 2 presents representative questions and activities from the data collection components.

**Table 2:** Data-collection components and representative questions.

Data-collection component	Representative questions
Individual journal activity	Do you discuss your sexual experiences with your family doctor or health care service provider? How does your cultural background and ethnic identity influence your sexual experiences and practices? Do you discuss aspects of your sexual activity with your family (or ethnic community)?
Sequential focus group session 1	What is your experience like being both a trans woman and a Middle Eastern young adult living in Ontario? When it comes to sexual health, sexual activity, and sexual practices, what issues do MENA youth living in Ontario face?
Take-home activity	To reflect on what we discussed in the first focus group, talk with friend group, look up on the internet, and identify or develop what you think is the most effective youth-driven strategy to promote MENA youth sexual health.
Sequential focus group session 2	What are some factors that affect access to sexual health support services for MENA youth in your community? What would you or MENA youth hope for or want to enhance accessibility to and experience with sexual health support services?
Additional mixed focus group	Who would be the main target audience for this intervention? Why is this intervention important, and what would some of the short-term or long-term objectives be? Who would be best suited to carry it out, such as a non-profit organization, educational institution, business, healthcare clinic, or media organization?

Following preliminary analysis of the 14 identity-based focus group sessions, an additional mixed focus group was held with 12 participants returning from across the earlier groups. In this session participants reviewed potential behavioral, structural and biomedical interventions identified through the earlier focus groups and the relevant literature and discussed whether and how these approaches resonated with their experiences, needs and priorities. They further elaborated on the proposed interventions by considering their intended audiences, objectives, potential delivery organizations and community partners, appropriate settings, anticipated implementation challenges, and desired short-term and long-term outcomes. Within each intervention type, participants ranked the proposed approaches according to their perceived relevance, importance, feasibility, and likelihood of meeting the needs of MENA youth. These rankings helped identify which interventions warranted further development. The purpose of this session was to deepen, prioritize and clarify the recommendations rather than to establish uniform agreement among participants.

**Figure 1:**
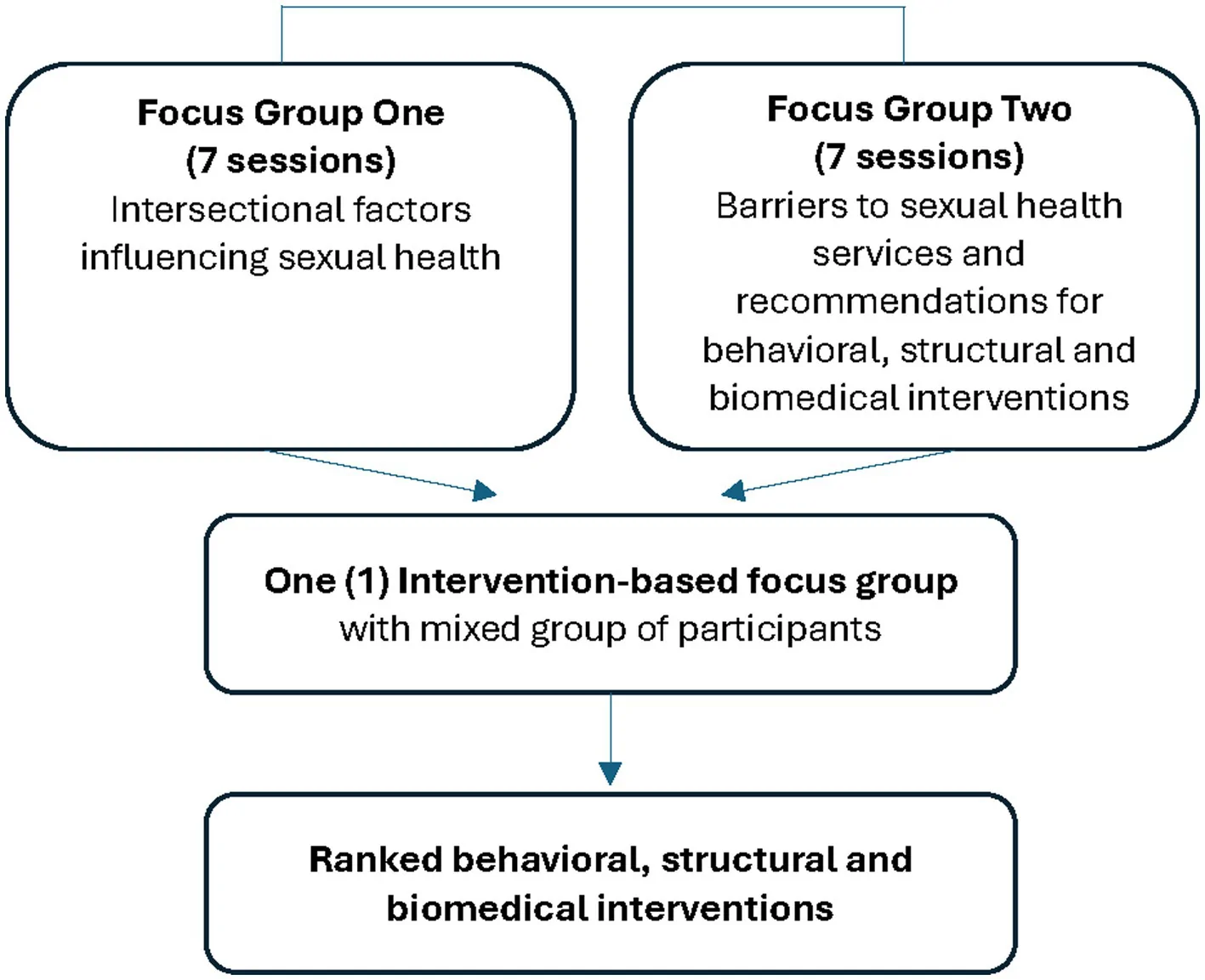
Breakdown of intervention-based data collection.

Figure 1 illustrates how the findings from the 2 sequential focus group sessions informed the additional mixed-group discussion and the development, refinement and prioritization of the intervention recommendations.

### Dataset adequacy

Data saturation was not used to determine the number of focus groups, recognizing that saturation is not a universal marker of quality across forms of thematic analysis (28). The number and sequence of groups were established in advance to support iterative discussion and further reflection on intervention recommendations through an additional mixed group with returning participants. Consistent with the concept of information power, sample adequacy was considered in relation to the study aims, sample specificity, richness and variation of participants’ accounts, and analytic strategy (29). The dataset was considered adequate because it included perspectives across the sexual and gender groups central to the study and generated sufficiently detailed and relevant accounts to address the research aims.

### Data analysis

This manuscript draws on the complete qualitative dataset, including all participant journals and focus-group discussions. Data were analyzed using codebook thematic analysis combining inductive and deductive elements (30). Broad forms of sexual health intervention introduced during the take-home activity informed the deductive analysis but were not treated as fixed analytic categories. Participants’ assessments, adaptations, rejections, and expansions of these approaches remained central to the analysis, ensuring that the resulting recommendations were grounded in their experiences and community knowledge.

Collaborative codebook thematic analysis was selected because it aligned with the study’s community-based approach and provided a shared but flexible structure through which academic researchers and PRAs could contribute to interpreting the data. CBR emphasizes co-learning, recognition of community knowledge and participation in knowledge and production (31). To support that emphasis, the first and second authors read and reread the transcripts to become familiar with the data and developed a preliminary codebook through close reading and discussion. The preliminary codebook was reviewed with PRAs, whose experiential and community knowledge informed revisions to the codes and their definitions. Differences in interpretation were discussed until consensus was reached. Following agreement on the preliminary codebook, the project coordinator coded the full dataset in NVivo, adding or refining codes when additional meanings or distinctions were identified. The project coordinator then organized the coded data into preliminary themes. PRAs and other members of the research team reviewed and refined the thematic structure through a collaborative visual sorting exercise. The final themes were agreed upon by consensus based on their internal coherence, distinctiveness, relevance to the study aims, and grounding across the dataset.

### Trustworthiness

Trustworthiness was considered throughout data collection and analysis in relation to credibility, dependability, confirmability, and transferability. During data collection, credibility was supported by giving participants several opportunities to reflect on and develop their perspectives. Participants first reflected privately through the individual journal activity, returned for a second discussion with the same group, and, for 12 participants, took part in an additional mixed focus group to further elaborate and refine the intervention recommendations. The private journal activity provided an opportunity to share perspectives outside the group setting, which may have reduced social desirability pressures when reflecting on sensitive sexual health topics. The sequential design also allowed participants to revisit earlier ideas, respond to the perspectives of others and identify areas of agreement and difference.

During analysis, dependability and confirmability were supported through the collaborative development and refinement of a shared codebook and review of the codes and themes by academic researchers and PRAs. Differences in interpretation were discussed as a team, and less common or divergent perspectives were retained where they contributed to understanding the range of participants’ experiences. Illustrative quotations were used to show how the interpretations were grounded in the data.

Transferability was supported by providing detailed information about the Ontario study context, participant eligibility and recruitment, the sexual and gender identity groups represented, the structure of the focus groups, and the data-collection and analysis processes. These details allow readers to assess whether the findings may be relevant to other populations and settings.

## Results

Participant characteristics are presented first to describe the composition of the sample. The remainder of the results presents themes generated from the qualitative data.

### Participant characteristics

Of the 56 youth who participated in the study, 65% identified their ethnicity as Middle Eastern, from countries such as Egypt, Iraq, Jordan, Lebanon, Palestine, Syria, Turkey, and UAE. The remaining 35% self-identified as Iranian, North African, multi-ethnic, or other. Most participants (79%) were born in countries in the Middle East and North Africa. Only 17% were born in Canada, which was reflected through newcomer experiences layered and intersected through the qualitative narratives. In terms of language, 62% spoke English and at least one other language (French, Arabic, Farsi, Kurdish, or other) at home. About half of the sample, 47%, indicated Islam as their religious affiliation, 20% indicated they were atheist or had no religious affiliation, 10% indicated they were Christian and 8% as agnostic. Other religious affiliations accounted for 15.7%.

In terms of sexuality, 39% identified as gay or MSM, 39% as either heterosexual male or female, 14% as trans women, and 7% as LBQ. Of the participants that indicated their gender identity, about 80% identified as cisgender (35.3% women and 43.1% men), 7.8% as trans women and genderqueer each, 3.9% as gender non-conforming men, and 2.0% as trans men. Most participants (65%) identified as single while 23.5% were in a relationship; however, 75% of participants indicated that they had been involved in some type of intimate relationship in the 6 months. Overall, 71% of participants had consensual sex in the past 6 months.

With regard to sources of sexual health information, the most commonly reported sources of information were friends at 62%; internet resources such as Google Search, Reddit groups, WebMD, and Mayo Clinic accounted for 51%; and health care practitioners accounted for 49%. The least popular sources of sexual health information (less than 5% each) were parents, teen sex info programs, anonymous phone lines, and youth groups. A majority of participants (88%) indicated having a family doctor, but only a small number had family doctors who are from the MENA community. Most participants, 76%, discussed their sexual experiences with their family doctors or regular health care providers.

While all participants discussed biomedical interventions and services such as PrEP, more than 50 % of the respondents (58%) did not know how often they should take PrEP (as currently recommended by the Canadian Guidelines). Only 22% of HIV-negative participants (12 respondents) indicated that they were taking PrEP, all constituted the gay and MSM or trans women groups. With respect to decisions about the use of PrEP, 73% of gay and MSM participants and 67% of trans women indicated they were likely to use it in future.

### Qualitative findings

**Figure 2:**
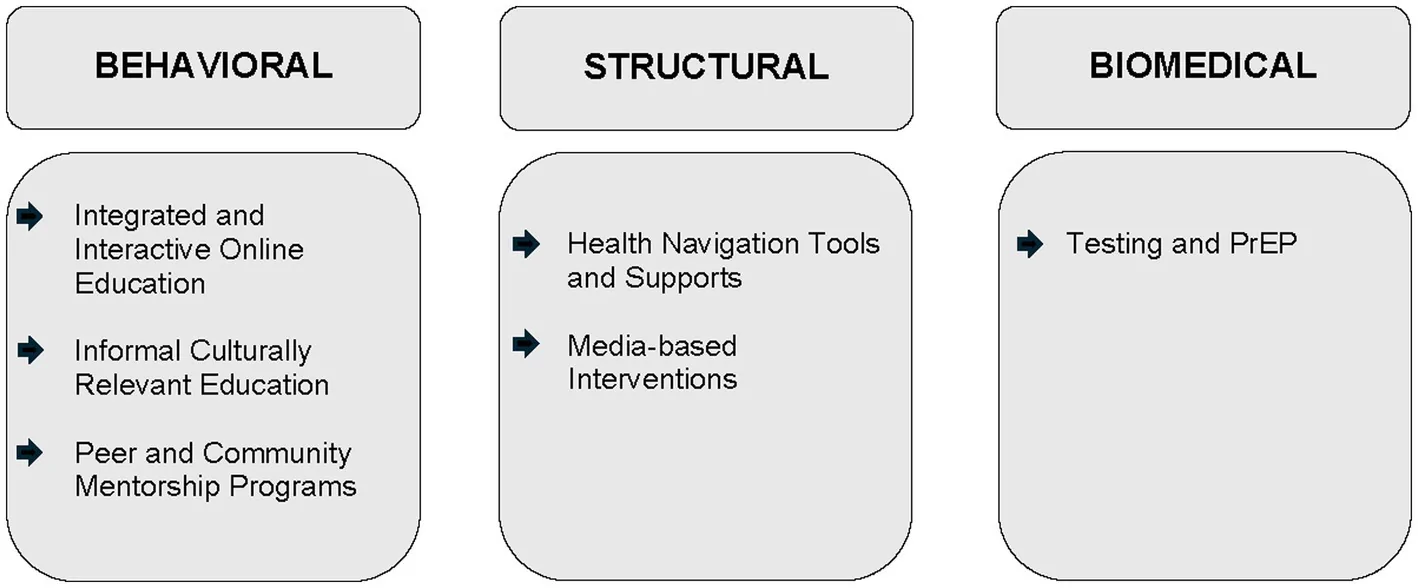
Key interventions as recommended by MENA youth.

The qualitative findings demonstrate the nuanced and intersectional nature of how youth engage with sexual health in terms of seeking information, sharing with friends and family, navigating sexual relationships, and accessing health services. The interventions recommended were informed by the complex realities of young people’s lives. In keeping with the focus group discussion, participants’ suggestions for health interventions were categorized under 3 broad areas, typical of sexual health interventions: behavioral, structural and biomedical (see Figure 2). Each of the sections below begins with the top-ranked intervention followed by the other key recommendations by MENA youth. Quotations are presented throughout without participant numbers or demographic identifiers to protect confidentiality and reduce the risk of deductive disclosure given the small, identity-specific composition of several focus groups.

### Behavioral interventions

#### Integrated and interactive online education

Within the category of behavioral interventions, online sexual health education integrated with social media and tailored to the MENA community either through language or other means of representation was the primary recommendation. The key objectives identified for this intervention were: to raise awareness around sexual health; empower people to gain knowledge; protect the health of self and others; and create a safe space online that represents the intersecting complexities of the MENA youth diaspora.

Participants overwhelmingly felt that there would be more meaningful uptake from the provision of resources online given the ease and convenience of access. *“I think you know, online platforms are definitely the way to go, I think they can really help to facilitate discussions on sexual health and sexual awareness there’s definitely a lot of potential in them.”*

Additional advantages of accessing resources online included those seeking sex education to be more discreet and anonymous. *“So a bunch of us agree that the online communities create a sense of safety, maybe or a sense of anonymity that a lot of us in the community crave.”* The anonymity was considered especially important given the taboo and sensitivity associated with conversations and education on sex and sexuality. *“The topic in general is just more taboo and maybe some people prefer to be anonymous or just like you know, have that safety behind a screen.”*

Participants suggested key considerations that would strengthen the provision of online resources, including language, interactive elements and integration across social media platforms. Many of the participants agreed that having information in languages common to the MENA region such as Arabic or Farsi would go a long way in increasing access, particularly for newcomer youth. “*I think there’s not enough tools and educational materials especially in Arabic, so I think including this as a part of an educational campaign would be beneficial.”* Ensuring that the resource be connected to social media and harness the potential of ‘influencers’ or social media celebrities was a popular suggestion as well. *“I always think people are influenced by other people through relationships, through awareness, through anything. That’s why I’m saying influence through a person, through the media.”* Additionally, it was suggested that instilling some formalized structure to these resources, such as a series of sexual health modules with a certificate of participation at the end, can incentivize and encourage participation.

Youth felt that an online hub for MENA youth on sex and sexuality could also be a critical source of support for youth who are not yet comfortable with their identities. *“When you are sort of in that stage of like, coming out or engaging in sexual activity, the first thing you do is to Google it and go to like certain websites, so I think it’s important to target those places.”* The suggestion for using the resource to springboard youth support groups facilitated online via Zoom was also discussed. To enable interaction participants suggested having a live chat agent who can guide them around the information available and connect them to other resources.

*Maybe if the topic, or the situation is so intense they can be like hey why do not we offer you a mentor or we can connect you to the right person and you can, you know, book a time to sit with someone and chat, depending on what your concern is, or like what you are trying to like figure out.*

Participants also suggested that the interactive element of the resource could potentially play a role in verifying sources of information to counter the spread of misinformation, a challenge that youth identified with searching for resources online.

#### Informal culturally relevant education

Participants frequently suggested initiating informal culturally relevant education programs. Informal education was considered distinct from formal learning in that its location can be diverse and is not held to a learning institution (i.e., recreation spaces, cafes, etc.) and its delivery is not dependent on certified teachers or experts:

*There are a bunch of organizations across the GTA that specifically deal with like Arab youth, and if those organizations could facilitate meetings, ‘like come hang out and let's talk about sexual health’, or bring in a doctor to talk about birth control to dispel some misconceptions and some myths, I think that would be wonderful.*

This covers a broad number of interventions, that based on participant input, can be further categorized into the following top three areas: language-specific education, gender-focused education, and family-centered education.

Under language-specific education, workshops in Arabic or Farsi delivered in community spaces or in partnership with relevant centers or groups were suggested. Participants not only raised language translations but also interpretation and the ongoing need for destigmatizing language used for words to convey ideas of sex, sexuality and/or gender:

*Some [newcomers] are scared to go [to health clinics] because of the language barrier, they wait for it, like they're waiting for someone else to come and go with them, but they can't actually talk to them about this kind of stuff also because of the taboo and stuff.*

Multilingual resources around these conventionally stigmatized topics can support overall education and awareness, connect people to relevant information and serve to destigmatize the issues.

Gender-focused education was a frequent recommendation made by groups with women-identified participants. The importance of educational content being both gender-inclusive and culturally relevant was highlighted, especially as a means to counter the pointed stigma around sex and sexuality placed on women. *“Ads are always targeted for women, like cisgender women. Women of the MENA region have like a certain other layer of stigma you know, it might help break down those barriers.”* Apart from the stigma, some women talked about how education needs to address the contextual realities affecting young diaspora women. *“Some Arab women begin sexual activity later in life because it’s so stigmatized and there’s some religious trauma so peer campaigns should not just be for 18 to 21 year olds, they can maybe be a little bit more inclusive.”* The need for education that addresses toxic masculinity or having spaces for men where they are supported to ask questions and demonstrate vulnerability was also marked as an important need:

*If I could do it, I would run a campaign for men that’s one part educational information, just throwing information their way. The second part is letting them ask questions, because I feel like a lot of them have so many questions not directly related to the reproductive system but, for example, about consent, intimacy, or connection, outside of all the macho pride nonsense.*

Lastly, within the area of informal education, family-centered education was repeatedly suggested across all the subgroups. This referred to education programs or resources tailored for families and delivered to parents, grandparents or youth. The importance of family in shaping young people’s perceptions of healthy relationships, sexuality and sexual health was raised in all groups and following from this, family-centered education served as a natural progression. *“I feel like that’s the biggest thing is the education awareness and breaking those stigmas and taboos, especially within family because, like, especially with MENA like we are always so like oriented with our family and community.”* Participants often discussed the value of education and awareness building with families to ensure that youth have safe and supportive environments at home when exploring their sex and sexuality:

*I think there needs to be awareness for parents. Hopefully we will become parents either by adoption or by birth whatever, but in my opinion we should speak to the parents, because they are the ones who are raising the children.*

The value of educational programs targeting families and situating content in a cultural context was shared by youth across all subgroups.

#### Peer and community mentorship programs

Community mentorship programs were a popular recommendation among many participants, especially within the cisgender women’s and gay and MSM groups. These initiatives were described as ones that would facilitate MENA community connection and support youth around sex and sexuality in a culturally relevant way:

*I just think it would be extremely helpful. Any form of advice for mentoring that I've ever gotten in terms of sexually related topics, has always been from the purely Canadian perspective and our experiences are quite different. So just having someone who could potentially have given me any advice or anything like that would have been very, very, very helpful at a younger age.*

Mentorship was seen outside of building expertise and/or sharing subject matter knowledge but more about sharing the lived experiences of people who represent ‘counter-narratives’ from what is most mainstream within the cultural community. It was also talked about as a source of education and learning from trusted friends and peers:

*There's also lack of supports, lack of mentors within a community where we can say you know, this is a MENA gay individual who is successful and has the life together, and this is someone I can turn to for support.*

Suggestions were typically framed around what participants would have liked to see when they were younger, how it informs ‘what I wish for youth to have now’, and peer initiatives supported by community and cultural organizations. *“There’s something very powerful about peer support and peer education, which is what a lot of the organizations are doing.”* Within this suggestion, the idea of what family-centered mentorship could look like was also raised to explore how parents and other family members may benefit from peer support models.

### Structural interventions

#### Health navigation tools and supports

Among the structural interventions recommended by youth, health navigation tools and supports were the predominant choice. Youth shared considerable challenges in navigating healthcare systems and services including barriers like lack of knowledge, language, racism, homophobia, and transphobia. Participants discussed the need for support when people are seeking help around their sexual health, such as accessing PrEP, screening, testing, and follow-up. “*If I had any issue, I do not know where I should go first, should I go to any clinic walk-in or I should go to the general hospital or where I should go.*” Given the personal nature of the services, participants noted the importance of having a trusting and safe relationship with the healthcare professional. *“So it would work like a vetted clinic or a group of trusted family doctors, gynecologists, urologists, whatever the case may be. Make sure that they are open minded enough or, you know, trusted enough.”* Additionally, participants referenced the confusion one might feel when trying to find information online or through peers/friends. It was important for participants that MENA diaspora youth, especially newcomers and/or youth accessing information for the first time, know about low-cost, easy access resources:

*I think it would be great if an organization put out something like here's a list of resources specifically for MENA youth like these are healthcare workers, therapists, these are nurses, social workers, that you can go to who have like specifically signed up to be there for you–I think that would be a really great resource.*

The key objectives for this intervention were identified as creating a resource or compilation of trusted, non-judgmental healthcare services and providers, encouraging queer and sex-positive spaces, training health care workers as required, enabling community members to sift through the plethora of health information available online, and increasing service access for those with MENA language needs. Some considerations in this area included resources for youth on how to navigate the health system such as lists of Arabic-speaking physicians or LGBTQ+ welcoming clinics:

*It would be useful to have just a list of doctors who are LGBTQ+ friendly and having that like some sort of tag or like if people put it on their offices so I know I'm conscious when I enter that space. When I see a rainbow flag or something I'm like oh cool so they're LGBT friendly.*

Health care workers who speak Arabic or other home languages from the MENA community were also considered highly valuable. *“Having a list of doctors who are not just LGBT friendly but can speak your language.”*

Training for health workers and/or interpreters on how they might respond to youth specifically around care needs around sex, sexuality and gender identity was a repeating area of focus:

*It becomes evident that you need competent healthcare workers that are aware of the issues needed by our community to be able to service our community. So there needs to be professional development courses offered to primary care providers to deliver culturally safe and appropriate care.*

The importance of training language interpreters was especially raised by newcomer youth across groups and the trans women’s group who highlighted training on anti-transphobia:

*Some people who are not very comfortable sharing or expressing themselves 100% in English, having a third person as a translator in a room can be inconvenient because you're not only expressing everything about your sexual experience to a single person you're expressing it to two people and one of them is not a medical professional, so that can be a thing for some people.*

Participants felt this intervention would be most helpful for newcomers who may have language barriers or fear judgment based on race, gender identity or sexuality:

*When you come into a new country it's always scary, no matter where you go it's always changing, so just making them aware of the resources they have available would really help them to make informed decisions and just feel more confident.*

Partnerships and collaborations of these trained multidisciplinary health teams with settlement agencies, LGBTQ+ centers, emergency services, language and interpretation schools, media centers, and social media influencers were suggested.

#### Media-based interventions

Health programs and projects situated within varying media platforms were repeatedly recommended across all subgroups. Participants mainly described media interventions as initiatives that focused on increased representation of MENA identities on mainstream platforms, challenging stereotypes and using social media to facilitate community connection and education. *“Just the idea of representation of just being able to see yourself or younger self on TV and seeing people having positive sexual experiences and safe sexual experiences would that be an effective intervention.*” Similarly, media interventions were also seen as a powerful tool for anti-stigma campaigns, especially around sexuality:

*I think media plays a huge role in in education and if we can somehow get more conversations happening at a larger scale in the media on Netflix shows, I think it goes a long way, like if you see a character, like a queer women.*

Social media was a significant subsection cited as necessary to reach people in the community with knowledge and experiential sharing. The use and potential of downloadable apps was also cited, specifically dating apps among the gay and MSM groups were discussed as pathways to promoting connection, education and support. *“Maybe podcasting is a great idea, or the WhatsApp campaign, which I think is a great idea, and yeah definitely social media, different social media platforms targeting different audiences.”* It was clear that different strategies would be needed for different groups.

Lastly, media and social media were described as having immense potential to facilitate community connection and bring people together in support of one another:

*I would love to see one day like a show in our MENA community, where we are discussing our issues and you know, raising awareness through a show that could go even global, not just in Canada. It could be, you know, like a voice to other people who are like in different parts of the world to know our stories, who could take it as an example, to feel they're not alone. More like an advocating platform.*

### Biomedical interventions

#### Testing and PrEP

The most frequently mentioned intervention in this category was the availability of testing and the different kinds of tests, such as home tests or clinic access. PrEP was also consistently referenced among the gay and MSM and trans women’s groups. *“PrEP would be an effective strategy but it does not prevent other STDs, for example, so you would also have to like get into using condoms and other systems.”* The open availability of sexual health medication and supplies (e.g., PrEP, condoms, HIV test kits) was identified as the most effective way to promote sexual health. Participants especially agreed that PrEP should be more accessible, especially on-demand, to people who might not be covered by OHIP or other insurance plans (i.e., it should cost less/no money and be as openly available to quickly order or pick up for free as condoms are). “*I think that the government also has to play a role, the prep that should be covered, that my opinion that for all people they they should not be just limited for people.”*

The need to normalize testing and health-seeking behavior regarding PrEP was highlighted as a crucial sexual health strategy to counter the fear of stigma and judgement. Participants suggested youth and/or newcomers should have access to kits with educational information and contact links and stressed that people should have the option of ordering safer sex supplies online:

*It would be nice if some people who might not live in the city or who don't have frequent visits to sexual clinics, if there was a way that they can subscribe to receive products online or something by mail and they would just pay for their shipment. I mean everything is now shifting online, we can't even go to a health clinic because of COVID and that's not preventing people from having sex, so the solution has to be just as radical as the problem.*

## Discussion

This study identified community-informed priorities for sexual health interventions for MENA youth in Ontario across diverse sexual and gender identities. The findings point to interconnected needs for privacy, culturally and linguistically relevant information, identity-affirming support, trusted pathways through healthcare systems, positive community representation, and affordable access to biomedical prevention. The intervention categories were used as organizing domains rather than as mutually exclusive classifications. Some recommendations crossed these boundaries because individual practices are shaped by the social and healthcare systems in which they occur. The following sections discuss these priorities across the behavioral, structural and biomedical intervention domains and consider their implications for developing responsive sexual health interventions for MENA youth.

### Behavioral interventions

Integrated and interactive online resources were participants’ primary recommendation. Previous research similarly suggests that youth find online educational resources safer and more accessible as they can ease potential anxiety, fear or embarrassment youth might face if accessing similar resources in person (19, 32). Reports by Population Reference Bureau on the sexual and reproductive rights of MENA youth specifically highlight the use of the internet to craft and disseminate sexual health education that would otherwise be difficult for youth to access (33). The report particularly discusses the increased use of mobile phones among youth in the region, further driving the potential of online and e-based interventions. For the diaspora youth in this study, the use of apps and social media interventions was cited as critical and is primarily accessible over phones. LGBTQ + youth especially indicate that online spaces often remove judgements that in-person services may apply to service users based on ethnicity, race or sexual identity (19, 34). Despite challenges related to data protection, privacy and technical access, online education targeting MENA youth is critical. Participants’ preference for online resources reflects a need for privacy, anonymity, and control over when and how sexual health information is accessed. Further, participants wanted resources that were multilingual, culturally responsive and interactive.

Informal education comprised the second key recommendation among behavioral interventions; specifically, education should be multilingual and culturally appropriate, gender-based, and family-centered. This is in keeping with research on the effectiveness of multilingual health education, as studies show that while translation is an important step, cultural interpretation and framing of health communication is equally critical (35). These findings suggest that multilingual intervention development should involve more than translating English materials into Arabic, Farsi or other languages. Youth and community members who understand how sexual health concepts are communicated, understood, or stigmatized within different communities should be involved in developing and reviewing the language. This may increase the relevance of the materials while avoiding the assumption that one translated resource will meet the needs of all MENA youth.

Gender-focused sexual health education tailored to the needs of cisgender women is well-established in the literature (36). Research shows that Arab women face particular pressures to maintain virginity and sexual innocence thus affecting their access to sexual health information and resources (37, 38). Youth in this study stressed the importance of interventions that account for women’s realities but they also recommended initiatives specifically targeting men. Research on sexual health education among young Iranian boys mirrors the importance of tailoring interventions for young boys given the gendered challenges they also face such as pressures to perform and not seek advice or help (39). Participants’ recommendations point to a need for education that addresses the gendered expectations shaping sexuality, including virginity, sexual respectability, masculinity, consent, intimacy, vulnerability, and help-seeking.

Family-centered education is also well studied in the broader literature; however, knowledge of interventions specific to racialized families in this regard is sparse. Research on family sex communication among the Arab American diaspora outlines how the challenges of stigma and lack of knowledge often impinge on parents’ ability to engage with their children on topics of sex and sexuality (40). These challenges were echoed among participants of this study as well; however, it is important to note that youth did not prefer parents as their source of sexual health information, their preference was simply to know that their families and parents in particular offered a safe and supportive space in case these conversations need to be had. This is understandable given the strong familial bonds highly valued by MENA communities (15, 41).

Finally, peer support models have gained traction as an effective and meaningful health intervention model, especially in the field of HIV education and prevention (42, 43). A key feature of peer support models is their potential to build on community experience, expertise and capacity in order to be effective (43). This is precisely what youth spoke of as a necessary component of their recommendation; for example, the mentorship described was not focused on professional expertise or networking but on community connection and the visibility of people in their community. Peer mentorship may therefore be valuable because it offers culturally situated recognition and makes diverse MENA sexual and gender identities more visible.

### Structural interventions

Under structural interventions, two main recommendations were outlined: tools to navigate the complexities of the health system and media-based initiatives situated in conventional or new media. The importance of initiatives and tools that support people in navigating health systems was in clear response to the significant barriers that youth spoke of when accessing health services. Barriers for newcomer youth in accessing varying systems in Canada and Ontario, including health and social care services, are well documented (44, 45). Research with Muslim youth in Toronto further outlines challenges in accessing sexual health information and services (13).

Participants across all the focus groups recommended tools that could help them identify where to go for healthcare professionals that are known to be inclusive, non-judgmental and knowledgeable. This is well-aligned with previous research (18, 46, 47). This suggests that the barrier is not always the complete absence of services, but uncertainty about which services are affordable, confidential, linguistically accessible, culturally responsive, and affirming of diverse sexual and gender identities. A navigation intervention would therefore need to be verified and regularly updated, with clear organizational responsibility, staffing, referral partnerships, and a process for responding when listed services do not meet expected standards. Youth also recommended that healthcare providers and language interpreters be trained to respond in non-judgmental and culturally responsive ways. These findings show that access involves more than obtaining an appointment. The quality and perceived safety of the interaction may affect whether youth disclose relevant information, return for care or seek services again. Training should therefore address confidentiality, cultural safety, anti-racism, sexual and gender diversity, anti-transphobia, and the privacy concerns that may arise when an interpreter is present during sexual health discussions.

The second key intervention recommendation in the structural category was related to media-based interventions, using either conventional or new media such as television or social media, respectively. Participants identified the usefulness of seeing positive representations of youth in their communities as a means of developing self-esteem and challenging harmful stereotypes, a common recommendation from racialized youth as indicated by existing research (6, 24). These findings suggest that representation may function as part of the intervention rather than only as a communication strategy. Seeing diverse MENA youth represented positively may reduce isolation, challenge stigma and strengthen community connection.

### Biomedical interventions

Key recommendations focused on PrEP and HIV testing. Specifically, youth from the gay and MSM and trans women’s groups recommended that PrEP be more widely available and affordable. Research on PrEP uptake among MSM and gay men in Toronto are aligned in the recommendation (48). While there is no research on the uptake of PrEP among MENA youth, research on the use of PrEP among other ethnocultural groups show that while awareness is high in all communities, Black and Hispanic men were less likely to talk to their physicians about PrEP or to begin using it (49). Consistent with earlier research (49), gay and MSM youth in this study demonstrated higher awareness of PrEP than other groups but talked about barriers to uptake and the need to normalize its usage. As a way to mitigate judgement from service providers, participants recommended increased accessibility to HIV self-testing kits. Research also shows that HIV self-testing kits offer strong opportunities to increase the uptake of testing, especially among vulnerable populations who experience barriers to health access (50).

### Implications for implementation

From an implementation perspective, participants’ recommendations speak most clearly to whether future interventions would be viewed as acceptable, appropriate, feasible, and accessible to MENA youth. Youth may be more likely to trust and engage with interventions that are culturally and linguistically relevant, protect confidentiality, affirm diverse identities, involve peers, and are delivered through trusted settings. At the same time, successful delivery will depend on practical support, including trained and adequately supported staff, appropriate technology, and strong community and organizational partnerships.

Since this study did not implement or evaluate the interventions proposed by participants, the findings should be understood as community-informed priorities rather than evidence that any intervention will be feasible, effective or sustainable. Future research should involve MENA youth and community partners in selecting and co-designing promising approaches and then assessing their acceptability appropriateness and feasibility through pilot implementation. Subsequent evaluation could examine organizational uptake, costs, reach, effectiveness, adaptations made during delivery, and longer-term sustainability.

Overall, meaningful involvement of MENA youth throughout the design, implementation and evaluation of any initiatives will be essential to ensuring that they reflect the diversity, priorities, and realities of their lives.

### Study limitations

Despite receiving the same training, the PRAs’ varied facilitation styles may have influenced the level and depth of discussion across focus groups. Moreover, the 2 focus groups that engaged trans and gender-diverse participants were conducted in Arabic due to language barriers. As such, there may have been some cultural nuances not captured in the discussions as a result of on-the-ground interpretation. Conducting the focus groups online may have limited participation among youth without reliable internet access, suitable technology or a private space. At the same time, the virtual format reduced geographic and travel barriers and enabled the participation of youth from different areas of Ontario, an important consideration when engaging geographically dispersed sexual and gender minority youth (51, 52).

Since participants self-selected into the study, youth who were more comfortable discussing sexual health or more connected to community networks may have been more likely to participate. The group setting may also have encouraged socially desirable responses, particularly when participants discussed sensitive topics or responded to the perspectives of others. In addition, the findings are situated within the Ontario context and reflect the study’s eligibility criteria, language requirements and participant composition. They should therefore not be understood as representing all MENA youth in Canada or the full diversity of MENA communities.

Finally, the positionalities and experiences of both the academic researchers and PRAs shaped the questions asked, the focus group interactions and the interpretation of the data. Collaborative analysis, reflexive discussion and attention to divergent perspectives were used to make these influences visible, although they could not be removed from the research process.

## Conclusion

The generation of applicable knowledge and literature driven by youth is crucial for crafting relevant, effective health interventions for the MENA youth diaspora in Ontario. As part of this study, MENA diaspora youth shared their stories and perspectives and provided significant knowledge to support the development of useful sexual health programs in the community. Tailored approaches that are culturally relevant, family-centered and gender-focused are identified as strong informal educational recommendations along with peer mentorship models and integrated online resources. Increasing health access and uptake by MENA youth through the development of health navigation tools is seen as a promising structural intervention. Media-based initiatives that promote positive community representations and serve to destigmatize sex and sexuality in the community are also prioritized and lastly, biomedical treatments such as PrEP and testing are considered crucial for strong health outcomes. Youth stressed the importance of all these interventions reflecting a fulsome understanding of sexual and gender diversity and being inclusive of LGBTQ+ MENA youth in practice. These key objectives identified through the study align with the existing literature while adding unique cultural perspectives as experienced by the MENA youth diaspora in Ontario. Knowledge generated through this study is an important step in building a solid body of evidence to drive effective sexual health interventions for Canada’s growing communities.

## Data Availability

The datasets presented in this article are not readily available because of identifying information in the transcripts that could compromise the privacy of research participants. Requests to access the datasets should be directed to the Youth Sexual Health and HIV/STI Prevention in Middle Eastern and North African Communities (YSMENA) Program Data Access Coordinator. Applicants should contact ysmenastudy@gmail.com through the YSMENA program site (home: https://www.ysmenaprogram.com/).
